# Detection of genomic mutations in blood and urine free circulating tumour DNA in patients with inoperable and metastatic lung adenocarcinoma harbouring an *EGFR* mutation in tissue: a UK pilot study

**DOI:** 10.3389/fonc.2023.1197037

**Published:** 2023-07-05

**Authors:** Helen Brooks, Ling Li, Alfredo Addeo, Megan Stevens, Charles Comins, Sebastian Oltean

**Affiliations:** ^1^ Bristol Haematology and Oncology Centre, Bristol, United Kingdom; ^2^ Department of Clinical and Biomedical Sciences, Medical School, Faculty of Life Sciences and Health, University of Exeter, Exeter, United Kingdom; ^3^ Oncology Department, University Hospital Geneva, Geneva, Switzerland

**Keywords:** lung cancer, EGFR, resistance to TKIs, CtDNA, plasma, urine

## Abstract

The development of methodologies to analyse circulating tumour DNA (ctDNA) in the blood or urine of cancer patients provides an invaluable resource that can be used for diagnosis and prognosis and to evaluate response to treatments. Lung cancer has seen in the last years a revolution in treatment strategy with the use of several classes of EGFR inhibitors. However, almost invariably, resistance to such therapies appears. In this paper, we describe a pilot, longitudinal study with 20 patients with confirmed *EGFR* mutations in tissue biopsy for lung cancer. The objective of the study was to determine whether ctDNA from plasma and/or urine could be used to monitor the *EGFR* mutational status of patients with confirmed *EGFR* mutation-positive non-small cell lung cancer (NSCLC) during treatment with EGFR inhibitors. Blood and urine were collected monthly over periods ranging from 6 to 16 months. CtDNA was analysed in each patient for the presence of several known mutations that predispose to resistance to EGFR inhibitors. We have proven that serial monitoring of ctDNA from both plasma and urine is feasible and that patients are willing to participate in this process. We have also shown that longitudinal ctDNA monitoring may detect resistance mutations before the development of radiological and clinical disease progression.

## 1 Introduction

Lung cancer is the most common cause of cancer death in the UK and worldwide (1, 2). There were an estimated 1.8 million deaths globally from lung cancer in 2020 (2). Around two-thirds of lung cancer deaths are attributable to tobacco smoking (2, 3). A molecular alteration is found in the majority of never smokers with non-small cell lung cancer (NSCLC), the most common being a mutation in *EGFR* gene (4). *EGFR* mutations are found in approximately 15% of patients with lung adenocarcinomas in Europe and are much more common in other parts of the world, with a frequency of nearly 50% in Asia and the Pacific (5).

In recent years, knowledge of the molecular sub-types of NSCLC has increased. Several actionable genetic alterations can now be identified, and targeted therapies have significantly improved survival for patients harbouring these mutations (6).

Routine testing for actionable mutations at diagnosis has become standard practice around the globe, often as part of a next-generation sequencing (NGS) panel. Tissue biopsy obtained from the primary tumour or a single metastatic site, for histological diagnosis of lung cancer, remains the gold standard. However, 10%–20% of biopsies obtain too little malignant tissue for immunohistochemical and genetic testing or fail to obtain malignant cells at all (7). Furthermore, a small fragment of tissue from a single site may not demonstrate a representative mutational profile owing to heterogeneity (8).

In-frame deletions within exon 19 (del19) account for 44% of all primary *EGFR* mutations. Single-nucleotide substitutions of arginine for leucine at codon 858 of exon 21 (L858R mut) account for a further 41%. The remaining 15% are made up of other single-nucleotide substitutions and in-frame duplications and/or insertions in exon 20, of which the latter are known to display primary resistance to EGFR inhibitors (9, 10).

EGFR-directed therapy leads to significantly improved survival compared with chemotherapy, with a more favourable toxicity profile (11–15). Despite initial good response to first-line tyrosine kinase inhibitors (TKIs), clinical progression inevitably occurs, commonly associated with the development of a resistance mutation. On progression with first- and second-generation TKIs, the T790M mutation can be found in approximately 60% of patients in tissue biopsy of a growing lesion (16). This is a single-nucleotide substitution in exon 20 of *EGFR* gene. For these patients, third-generation TKI osimertinib has demonstrated a significantly increased progression-free survival when compared to standard second-line chemotherapy (17).

At the time of disease progression, tissue biopsy from a progressing site is not always technically possible or acceptable to the patient. Once resistance to a TKI develops, clinical deterioration can occur rapidly, so identification of driver mutations needs to occur quickly. Molecular analysis at progression is also needed to increase our knowledge of other resistance mechanisms to support the development of novel therapies.

Free circulating tumour DNA (ctDNA) could be used to overcome many of these problems. CtDNA has most commonly been obtained from blood (plasma or serum) but can be obtained from other bodily fluids such as urine, cerebrospinal fluid, and saliva (18). Obtaining samples is minimal/non-invasive and can be performed promptly in the clinic setting. The *EGFR* mutation has successfully been identified on ctDNA in patients known to have an *EGFR* mutation in tissue (18–27).

CtDNA is released from both the primary tumour and metastases, providing a representative mutational profile and overcoming heterogeneity (28). Regularly analysing the ctDNA mutational profile during treatment may potentially allow the identification of resistance mutations ahead of clinical progression.

Previous, mostly retrospective, studies have shown that ctDNA can be used for the detection of *EGFR* mutations. Here, we report our data on the prospective validation of droplet digital polymerase chain reaction (ddPCR) as a method of identifying *EGFR* mutations from plasma and urine, from diagnosis to progression, in patients treated with first- and second-generation TKIs. This would potentially allow a move from invasive tissue biopsy to minimally invasive blood sampling or non-invasive urinary testing to identify resistance mechanisms and guide treatment.

## 2 Materials and methods

### 2.1 Study design

This is an observational, non-interventional pilot study. A total of 20 patients with locally advanced, inoperable, or metastatic NSCLC with an *EGFR* mutation detected in tissue were treated with first-line first- or second-generation TKI as the standard of care at the time of the study. The study recruited across two Bristol Hospitals: the Bristol Cancer Institute (University Hospitals Bristol) and Southmead Hospital (North Bristol NHS Trust).

The study was conducted in accordance with the Declaration of Helsinki and the International Council for Harmonisation Good Clinical Practice standards. Approval from NHS Research Ethics Committee and Health Research Authority was received at each study site. The study is sponsored by the University of Exeter and funded by Boehringer Ingelheim.

The objective of the study is to determine whether ctDNA from plasma and/or urine could be used to monitor the *EGFR* mutational status of patients with confirmed *EGFR* mutation-positive NSCLC during treatment with an EGFR TKI.

*Primary endpoint*: to assess whether ctDNA extracted from plasma and/or urine could be a reliable source of *EGFR* testing.

*Secondary endpoint*: to assess whether longitudinal monitoring of ctDNA could be used as a prognostic indicator of clinical or radiological progression of disease during EGFR TKI therapy.

### 2.2 Participants

A total of 20 patients were recruited between December 2017 and June 2019, when the recruitment target was met. Patients were identified through screening all genetic reports produced by the Bristol Genetics Laboratory from all lung cancer analyses. Every consecutively diagnosed patient across both sites was approached to consider participating in the study until the target of 20 patients was achieved.

The inclusion and exclusion criteria are detailed in **Table 1**, and participant characteristics are displayed in **Table 2**.

**Table 1:** Study inclusion and exclusion criteria.

Inclusion criteria
Age 18 or over
Histologically confirmed adenocarcinoma of the lung with an EGFR mutation
Inoperable or metastatic (stage IIIB–IV) disease
Treatment naive in the advanced stage setting (prior chemotherapy in the adjuvant setting was permitted)
Fit to receive afatinib (or other TKIs if deemed unsuitable for afatinib)
Willingness to participate and provide monthly blood and urine samples
Exclusion criteria
Unfit for standard EGFR TKI therapy
EGFR exon 20 mutation

TKIs, tyrosine kinase inhibitors.

**Table 2:** Participant baseline characteristics.

Sex	*Number (%)*
Female	18 (90)
Male	2 (10)
Age	*Years*
Mean	69.4
Median	71.5
Range	44–82
Ethnicity	*Number (%)*
White British	19 (95)
Asian Pakistani	1 (5)
ECOG performance status	*Number (%)*
0	6 (30)
1	11 (55)
2	2 (10)
3	1 (5)
Stage	*Number (%)*
IIIA	2 (10)
IIIB	0
IIIC	0
IV	18 (90)
Smoking status	*Number (%)*
Never smoked	6 (30)
Stopped smoking >20 years ago	9 (45)
Stopped smoking 10–20 years ago	4 (20)
Stopped smoking <10 years ago	1 (5)
Current smoker	0
EGFR mutation type in tissue at baseline	*Number (%)*
Deletion 19	10 (50)
L858R	7 (35)
Deletion 19 and T790M	1 (5)
Exon 18 c.2156G>C	1 (5)
Exon 21 c.2582T>A	1 (5)

ECOG, Eastern Cooperative Oncology Group.

Afatinib (or first-generation TKI at clinician’s discretion) was prescribed and administered according to the European Summary of Product Characteristics.

### 2.3 Assessment

Patients attended for clinical review and blood and urine sampling at baseline, 2 and 4 weeks after commencing treatment, and four-weekly thereafter. Sample collection was ceased when first-line TKI was stopped (for progression of disease, toxicity, or patient choice) or on withdrawal of consent. Blood measuring 15–20 ml was collected (to produce 10 ml of plasma) in sample tubes containing ethylenediaminetetraacetic acid (EDTA), and 50 ml of urine was collected in a plain tube. Computed tomography (CT) scans were performed at intervals of 3 months as standard of care.

Outcome data were collected beyond the termination of sample collection, including overall survival data.

At the point of progression, if appropriate, patients were offered repeat tissue biopsy as per standard of care, specifically looking for the development of the T790M resistance mutation.

All serious adverse events and serious adverse reactions during TKI treatment were reported to the sponsor and Boehringer Ingelheim.

### 2.4 DNA extraction and mutation detection

Plasma was extracted from the blood samples, and the cellular component of the urine samples was removed by centrifugation. CtDNA was extracted using the Qiagen QIAamp Circulating Nucleic Acid Kit, as per the manufacturer’s guide. All samples were tested for *EGFR* deletion 19, L858R, and T790M mutations.

TaqMan probes specific for each of the three mutations were used. Samples were fractionated into 20,000 droplets, and the PCR was undertaken in each droplet. The presence or absence of mutation in each droplet allows for quantification of the mutation present in the sample as a whole (29).

## 3 Results

Baseline plasma samples were collected for all 20 patients. One patient (del19) was unable to provide a baseline urine sample. Following the completion of enrolment, it was decided by the investigators to exclude the patient with exon 18 mutation from analysis, given that this mutation was not examined by the ddPCR testing employed here. One patient withdrew consent within the first 2 weeks of the study and so was not included in the analysis. Baseline samples for the remaining 18 patients were analysed and used to calculate true-positive and true-negative results, compared with tissue samples, to inform the feasibility of a future larger-scale study. Five patients remained in the study for 12 months or more, and their molecular profiles over time were examined.

### 3.1 Baseline analysis

#### 3.1.1 Deletion 19

On baseline ctDNA testing of the 10 patients with an exon 19 deletion in tissue, four were confirmed positive on plasma (n = 4/10) and none on urine (n = 0/9). Plasma ctDNA testing, therefore, identified four true-positive del19 and reported six false negatives. Urine ctDNA did not identify any true-positive del19 and reported nine false negatives.

Of the eight patients with non-del19 (L858R, exon 21), plasma ctDNA identified seven true-negative del19 (n = 7/8) and reported one false positive. Urine ctDNA also identified seven true negative del19 mutations (n = 7/8) and reported one false positive. The false positives from plasma and urine were from different patients, and therefore, six out of eight patients demonstrated complete concordance of negative results in tissue, plasma, and urine (**Table 3**).

**Table 3:** Del19 baseline ctDNA testing.

Del 19		Tissue	
		+ (n = 10)	− (n = 8)
Plasma ctDNA	+	4	1
	−	6	7
Urine ctDNA	+	0	1
	−	9	7
	No sample	1	**/**

ctDNA, circulating tumour DNA.

Plasma ctDNA demonstrates a sensitivity of 40% and a specificity of 87.5% compared to baseline tissue testing. Urine ctDNA demonstrates a specificity of 87.5%. There were no positive urine ctDNA results in the tissue-positive group, and therefore, sensitivity is zero.

#### 3.1.2 L858R

Baseline plasma ctDNA testing of the seven patients with L858R mutations in tissue identified five true-positive L858R mutations (n = 5/7) and reported two false negatives. Urine ctDNA identified two true-positive L858R mutations (n = 2/7) and reported five false negatives.

Of the 11 patients with non-L858R mutations in tissue (del19, exon 21), plasma ctDNA identified nine true-negative L858R mutations (n = 9/11) and reported two false positives. Urine ctDNA identified five true-negative L858R mutations (n = 5/11) and reported five false positives.

The two patients with positive urine ctDNA L858R were also positive on plasma and, therefore, demonstrated complete concordance of positive results across tissue, plasma, and urine. Five patients who were negative on the tissue for L858R revealed a negative result on plasma and urine, demonstrating complete concordance (**Table 4**).

**Table 4:** L858R baseline ctDNA testing.

L858R		Tissue	
		+ (n = 7)	− (n = 11)
Plasma ctDNA	+	5	2
	−	2	9
Urine ctDNA	+	2	5
	−	5	5
	No sample	**/**	1

ctDNA, circulating tumour DNA.

The sensitivity for L858R mutation testing on plasma ctDNA compared with tissue is 71.4%, with a specificity of 81.8%. Urine ctDNA has a sensitivity of 28.6%, with a specificity of 50%.

#### 3.1.3 T790M

It was very unusual that one patient was found to have a T790M mutation in tissue at baseline. On plasma ctDNA for this patient, a T790M mutation was identified, but it was not identified on urine ctDNA. Plasma ctDNA identified nine true negative T790M mutations (n = 9/17), and urine ctDNA identified 13 true negatives (n = 13/17) (**Table 5**).

**Table 5:** T790M baseline ctDNA testing.

T790M		Tissue	
		+ (n = 1)	− (n = 17)
Plasma ctDNA	+	1	8
	−	0	9
Urine ctDNA	+	0	3
	−	1	13
	No sample	/	1

ctDNA, circulating tumour DNA.

Given that there is only one positive tissue result for reference, it is not possible to calculate sensitivity. The specificity for T790M testing on plasma ctDNA is 52.9%, and for urinary ctDNA, it was 81.3%.

### 3.2 Molecular profiles over time

#### 3.2.1 Plasma ctDNA

**Figure 1:**
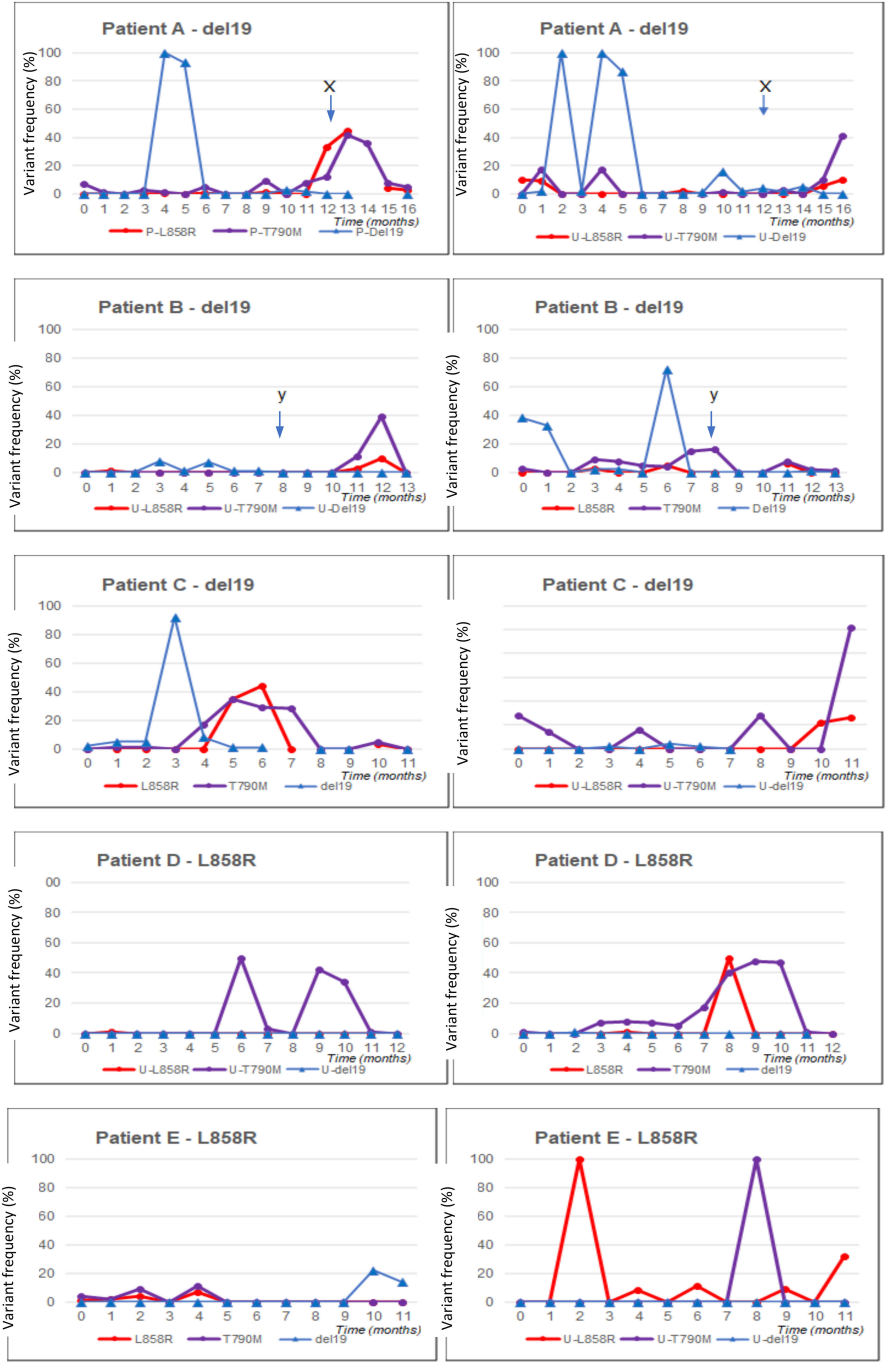
Blood and urine ctDNA analysis for five patients with long follow-up (11–16 months). Panels are shown for each patient (denoted as **A–E**). On the left, the blood profile is shown; on the right, the urine profile. ctDNA, circulating tumour DNA.

Five patients remained on treatment long enough to collect 11–16 months of data. Their molecular profiles for the duration of the treatment using plasma ctDNA have been reviewed in detail (**Figure 1**).

##### 3.2.1.1 Patient A

Patient A had an exon 19 deletion in tissue biopsy. The disease was confined to the thorax. The patient was on afatinib for 13.5 months. CT scan after 3 months of therapy demonstrated a partial response. Afatinib was stopped when the patient developed acute bowel perforation due to a perforated diverticulum. The disease remained stable off treatment at the data cut-off.

The levels of L858R and T790M mutation on plasma, undetected and present at low levels at baseline, respectively, increased dramatically at the point when TKI therapy was stopped.

Although deletion 19 was not reliably detected throughout the serial analysis, including no detection at baseline, it was detected at very high levels on two occasions.

##### 3.2.1.2 Patient B

The patient had deletion 19 in tissue biopsy. This patient had brain, bone, and lymph node metastases at diagnosis. After 3 months of treatment, there was a response in the brain and bones. After 8 months of afatinib, there was a symptomatic progression of a single brain metastasis. Shortly preceding this, there was a high level of deletion 19 detected and a significant rise in T790M mutation detection in plasma. T790M was found at a very low level at baseline. The metastasis was treated with Gamma Knife radiotherapy, and afatinib was continued. T790M and deletion 19 levels in plasma returned to being undetectable. The disease was stable at the data cut-off.

Although deletion 19 was not reliably detected throughout the serial analysis, it was detected at very high levels on three occasions. These were at baseline and month 1 and shortly preceding a disease progression event.

##### 3.2.1.3 Patient C

Patient C had deletion 19 in tissue biopsy. The disease was confined to the thorax. CT scan after 3 months of treatment demonstrated a partial response. After 9 months of afatinib, the disease began to progress. T790M mutation was not detected on local plasma testing or repeat tissue biopsy. No mutations were detected at or after this time on study plasma testing.

Although deletion 19 was not reliably detected throughout the serial analysis, it was present at low levels in the first 3 months and detected at a very high level on one occasion.

##### 3.2.1.4 Patient D

Patient D had an L858R mutation in tissue biopsy. The disease was confined to the thorax. The L858R mutation was not detectable in plasma at baseline. CT scans at 3 and 6 months demonstrated a partial response to treatment, and the disease was stable at the data cut-off. Low levels of T790M mutation were identified from month 4, and this then rose further in months 8 to 11. L858R also showed a sharp peak in month 9.

##### 3.2.1.5 Patient E

Patient E had an L858R mutation in tissue biopsy. The disease was confined to the thorax. CT after 3 months of afatinib showed a partial response, and the disease remained stable at the data cut-off.

#### 3.2.2 Urine ctDNA

There is minimal correlation between plasma and urine ctDNA testing. However, all five profiles examined demonstrated a rise in T790M mutation on urine testing towards the end of the data collection period. This may indicate that urine ctDNA is a reliable method of sampling to identify the T790M resistance mutation, and further examination is required.

## 4 Discussion

### 4.1 *EGFR* mutation detection in plasma ctDNA

#### 4.1.1 Deletion 19 detection on plasma ctDNA

The deletion 19 ctDNA assay employed here failed to demonstrate reliable detection of deletion 19. This may be due to the fact that deletions within exon 19 are heterogeneous and do not represent a single-point mutation.

Thress et al. (2015) also reported difficulties with exon 19 deletions on ddPCR, finding that they could only detect a minority of the known exon 19 deletion. They discounted ddPCR as a method from further analysis on the basis of this, despite it being a highly sensitive method for detection of the other mutations (22). Xu et al. (2017) did however successfully use ddPCR to identify all three of the common mutations (24).

#### 4.1.2 L858R mutation detection on plasma ctDNA

In this small sample, L858R mutation ctDNA plasma detection demonstrated good concordance with tissue results. The assay appears to be reliable. It will be of interest to examine a larger cohort of patients based on this pilot result.

#### 4.1.3 T790M mutation detection

For the one patient carrying a T790M mutation in tissue, this was correctly identified on plasma ctDNA. Surprisingly, T790M mutation was identified in plasma ctDNA in eight out of 17 patients who did not demonstrate the mutation in tissue. It is possible that these are false-positive results, as a result of an unreliable assay or small sample size. However, this may be a genuine mutation present in plasma, representing tumour heterogeneity with this mutation present at metastatic sites, in tumour cells, which are likely to be biologically different to those at the primary site, in order for them to have metastasised.

Of the eight patients with T790M present in plasma at baseline, six had an extra-thoracic disease, and the remaining two had a disease confined to the thoracic cavity. Of the seven patients who were plasma negative for T790M at baseline, five had disease confined to the thoracic cavity, with only two having an extra-thoracic disease. The observation that T790M mutation is detected more frequently in plasma at baseline in patients with an extra-thoracic disease than in those with disease confined to the thorax has been reported previously (23). There was no correlation in this small pilot study between T790M mutation in plasma ctDNA at baseline and shorter time to progression, but it will be of interest to report this in a larger study.

### 4.2 *EGFR* mutation detection in urinary ctDNA

Detection of *EGFR* mutations in urinary ctDNA was poor. CfDNA in plasma is thought to be protected from degradation by extracellular vesicles or nucleoprotein complexes, which are vectors in plasma that carry molecules or specifically DNA, respectively. CtDNA is excreted into urine from the bloodstream following glomerular filtration by the kidneys. CfDNA undergoes cleavage by urine nucleases as it passes through the kidneys, resulting in highly fragmented ctDNA entering the urine. These small fragments at low abundance are difficult to detect (7).

Franovic et al. (2017) overcame these challenges of detection in a method similar to that described by Reckamp et al. and Husain et al. (7, 26) by combining short-footprint mutation enrichment PCR with NGS. They demonstrated sensitivity and specificity of 94% or greater for the detection of the three most frequent *EGFR* mutations from urine ctDNA (30).

Li et al. (2017) and Hu et al. (2018) however both reported high sensitivity and specificity in obtaining *EGFR* mutations from urinary ctDNA using ddPCR alone (31, 32). Hu et al. used EDTA to preserve the urine once collected. Reckamp also reported using a preservative in the urine samples in their dual-method analysis.

Reckamp et al. reported increased sensitivity with greater volumes of urine collected, from 67%–75% for all volumes to 80%–93% with volumes of 90–100 ml (7).

### 4.3 Tumour heterogeneity

Similar to the findings in this study, Jenkins et al. (2017) also reported a higher discordance between the detection of T790M mutation in tissue and plasma, both in patients with an extra-thoracic disease and those receiving third- or greater-line treatment. They too hypothesised that tumour heterogeneity may be responsible, with increasing mutations developing at different metastatic sites, with advancing disease and in the presence of increasing lines of treatment (23).

In an analysis of circulating tumour cells (CTCs) by Maheswaran et al. (2008), the T790M mutation was unexpectedly identified in 10 out of 26 patients at diagnosis (prior to TKI therapy). Although patients carrying this mutation in CTCs still responded to first-generation TKIs, this mutation was found to be associated with a significantly reduced progression-free survival of 7.7 versus 16.5 months (33).

The theory that tumour heterogeneity is responsible for the identification of *EGFR* mutations in ctDNA not present in tissue biopsy was proven by Wan et al. (2017). They found a discordance of primary *EGFR* mutation positivity between tumour tissue and ctDNA, with up to 10% of patients testing negative for *EGFR* mutation in tissue, having an *EGFR* mutation identified on ctDNA. DdPCR has been shown to be a more sensitive method for detecting *EGFR* mutations on ctDNA compared with amplification-refractory mutation system (ARMS) (22). Wan et al. found more ctDNA *EGFR* mutations in tissue-negative patients with the more sensitive ddPCR compared with ARMS. Further review of EGFR tissue-negative patients with positive ctDNA revealed that a significant proportion of these patients had the typical characteristics associated with *EGFR* mutations, for example, never smokers and female sex. This led the investigators to conclude that an *EGFR* mutation is potentially missed in up to 10% of tissue biopsies due to heterogeneity and may be confirmed on plasma ctDNA using highly sensitive methods such as ddPCR. They confirmed the presence of intratumoral heterogeneity by analysing the pathology of multiple different foci of tumour from a sample of resection specimens (34).

The flip side to using ctDNA to overcome tumour heterogeneity is the potential to miss the driver resistance mutation by dilution. When the disease is progressing at only one site, in order to identify a resistance mutation, a biopsy should be taken from this area, as it is these cells specifically that have developed resistance (16). If the area of progression is small in terms of overall disease burden, and ctDNA is used, although the resistance mutation may be present in ctDNA from tumour cells at the progressing region, the volume of ctDNA from this area may be very small in terms of overall ctDNA volume with ctDNA from the rest of the tumours, and the resistance mutation, therefore, may be overlooked. This is a hypothesis and needs further validation.

### 4.4 Longitudinal ctDNA monitoring

In the five patients who remained on treatment for 12 months or more, the mutation load of T790M and L858R appears to increase significantly at approximately 9 to 12 months of treatment. T790M is a common resistance mutation, and its emergence at around this time point would be in keeping with the development of drug resistance, leading to treatment failure at an expected median time of 10 to 12 months. It has been demonstrated that levels of the primary mutation are suppressed with effective treatment, and levels increase again as treatment fails (35). This principle would account for rising levels of an L858R mutation in the patient who had an L858R mutation in tissue biopsy at baseline. However, L858R levels increased significantly in all four patients, suggesting that this mutation may develop as a resistance mutation. To our knowledge, this has not been reported in the literature.

In a poster presented at ASCO 2019, Zhou et al. demonstrated that clearance of the *EGFR* driver mutation, defined as having an undetectable mutation on ctDNA when the mutation was detectable at the outset, at 3 and 6 weeks post initiation of TKI, was associated with longer progression-free survival as compared with having a detectable mutation (36). It is possible, therefore, that those patients having a poor molecular response could be monitored more closely or have treatment changed or intensified.

### 4.5 Limitations of this study

This work was undertaken as a small pilot study to assess the feasibility of conducting a larger study. Though statistical analysis has been reported here, this is likely to be unreliable given the very small sample size. The trends and observations remain of interest, particularly when similar observations have been reported from other studies.

Shortly following the conclusion of this study, third-generation TKI osimertinib became the standard of care in the first-line setting. Therefore, the detection of T790M mutations during treatment with first- or second-generation TKIs is no longer a clinically important finding. Resistance to osimertinib is more heterogeneous. NGS is also now increasingly available, and the use of this technology makes identifying a range of alterations easier. Future work is, therefore, likely to use NGS rather than ddPCR, but many of the principles and finding for this study would still help to inform any future work in this area.

### 4.6 Conclusions

*EGFR* mutation detection in ctDNA has many potential important roles in the era of precision medicine. As knowledge of driver and resistance mutations increases, and new drugs are developed to target these molecular alterations, the need for a minimally invasive, reliable, and rapid test is paramount. This small pilot study was designed to test the reliability of using ddPCR to detect ctDNA from plasma and urine and the feasibility of carrying out serial ctDNA monitoring and to identify any issues that can be rectified before undertaking a larger study.

It has highlighted the difficulties in detecting deletion 19, in addition to suggesting that ddPCR is a reliable method of testing for the L858R mutation. It has informed the theory that the T790M mutation is commonly found at baseline in plasma, but not in tissue. It has proven that serial monitoring of ctDNA from both plasma and urine is feasible and that patients are willing to participate in this process. It has also shown that longitudinal ctDNA monitoring may detect resistance mutations before the development of radiological and clinical disease progression.

In conclusion, the use of ctDNA is a rapidly evolving area, with a myriad of research taking place. This pilot study shows potential for further development, in addition to some limitations that need to be overcome before designing a larger study.

The recently reported EORTC APPLE study demonstrated that during treatment with first-line EGFR TKI gefitinib, serial monitoring for T790M mutation led to the detection of “molecular progression”, i.e., development of T790M mutation on ctDNA ahead of progressive disease on imaging, in a clinically significant number of patients. The study suggests that switching these patients to osimertinib at molecular progression correlated with improved progression-free survival (37).

## Data availability statement

The raw data supporting the conclusions of this article will be made available by the authors, without undue reservation.

## Ethics statement

The studies involving human participants were reviewed and approved by Health Research Authority, UK. The patients/participants provided their written informed consent to participate in this study.

## Author contributions

HB, AA, CC, and SO contributed to the conception and design of the study. HB and CC collected the patient samples. LL and MS performed the sample analysis. HB wrote the first draft of the manuscript. All authors contributed to the article and approved the submitted version.
